# Repeatability of feed efficiency and its relationship with carcass traits in Hanwoo steers during their entire growing and fattening period

**DOI:** 10.5713/ab.24.0074

**Published:** 2024-04-25

**Authors:** Hyunjin Cho, Kyewon Kang, Hamin Kang, Seoyoung Jeon, Mingyung Lee, Eunkyu Park, Seokman Hong, Seongwon Seo

**Affiliations:** 1 Division of Animal and Dairy Sciences, Chungnam National University, Daejeon 34134, Korea; 2 Woosung Feed Co., Ltd., Daejeon 34379, Korea

**Keywords:** Carcass Trait, Feed Conversion Ratio, Feed Efficiency, Hanwoo, Residual Feed Intake

## Abstract

**Objective:**

This study investigated the repeatability of feed efficiency and its association with carcass traits in Hanwoo steers during the entire growing and fattening periods.

**Methods:**

The growth and intake of thirty-six Hanwoo steers (259±19.7 kg; nine months) were monitored throughout five periods, including two growing periods (GP) and three fattening periods (FP). The steers were fed two types of concentrate mixes with varying nutrient compositions until they reached a target weight of 800 kg for slaughter. For each period, steers were categorized into three classes based on their feed efficiency rankings using residual feed intake (RFI) and feed conversion ratio (FCR). Feed efficiency repeatability was assessed using the Spearman correlation coefficient, decomposition of random errors, and the Theil segregation index (TSI). The Pearson correlation coefficient was used to examine the relationship between feed efficiency and carcass traits.

**Results:**

The results demonstrated a significant and high correlation with RFI, but not FCR, during the growing or fattening stages (r>0.5; p<0.01). When steers were classified according to their feed efficiency rankings, 58% of the animals in the high RFI class (low efficient) initially (GP 1) remained in the same class by the last period (FP 3), whereas steers were randomly distributed based on FCR. The repeatability, assessed by the decomposition of random errors, was higher for RFI (0.61) than for FCR (0.15). The TSI also indicated that RFI rankings, rather than FCR rankings, are more likely to be maintained. Moreover, a weak association was observed between feed efficiency and carcass traits.

**Conclusion:**

In conclusion, RFI repeatability throughout the GP and FP surpassed that of the FCR, with steers classified as high RFI during the GP more likely to remain in the same class during the FP. Feed efficiency was weakly correlated with carcass traits.

## INTRODUCTION

One of the primary goals of beef producers is to maximize profitability by reducing production costs and increasing product value (i.e., improving carcass traits). As feed is the largest and most variable expense in beef production [1], improving animal feed efficiency is critical for increasing farm profitability. In addition, beef cattle with favorable feed efficiency can positively affect the environment by reducing methane and manure emissions [2,3].

Feed efficiency in beef cattle has traditionally been measured as the ratio of body weight (BW) gain to consumed feed (G:F) or its inverse (F:G or feed conversion ratio [FCR]; [4]). Feed conversion ratio is moderately heritable and is negatively correlated with average daily gain (ADG) and mature body size [5]. Another common method for measuring feed efficiency in cattle is residual feed intake (RFI), as described by Koch et al [6]. Residual feed intake in beef cattle is commonly defined as the difference between the actual and predicted intake based on ADG and metabolic BW (BW^0.75^; MBW). Residual feed intake is known to be moderately heritable, independent of ADG and mature size [1]. In general, the feed provided to animals in a beef production system has different characteristics and nutrient contents depending on the productive stages (e.g., growing and fattening periods). Additionally, there may be differences in the contents of nutrients in the feed even at the same stage (e.g., growing or fattening stages) of production. As a result, for FCR and RFI to be reliable indices for measuring an animal’s feed efficiency, the rankings of animals based on the index in a herd need to be correlated (or repeated) throughout the productive and physiological stages, although the exact values may change [7].

A few studies have tested the repeatability of feed efficiency, primarily based on RFI, and have shown that feed efficiency can be repeated at various stages of growth in cattle. Kelly et al [8] found that when beef cattle were fed the same diet for two separate periods of 114 d, each with a five-month interval between the two periods, the RFI was moderately repeated (r = 0.62). Similarly, RFI was moderately repeated (r = 0.40) by Gomes et al [9], in which the same diet was provided to beef cattle for two separate periods (period 1 of 70 d and period 2 of 134 d with a 21 d interval). Residual feed intake was also moderately repeated even when the feed was switched from a growing diet to a finishing diet in steers [10] and heifers [11]. Durunna et al [10] also indicated that FCR could be repeated and demonstrated that the class of steers based on the ranking of FCR was more repeatable when fed the same diet than when switched from a growing diet to a fattening diet (43.7% vs 38.4%). However, no study has investigated the changes in the repeatability of feed efficiency in cattle throughout the entire feeding period, which includes both the growing and finishing stages, especially in an intensive feeding system where a priori-determined amount of high-concentrate diet is provided based on a feeding program. Moreover, to the best of our knowledge, no previous studies have determined the repeatability of feed efficiency in Hanwoo steers.

Feed efficiency may also be related to carcass traits (i.e., carcass weight [CWT], backfat thickness [BFT], ribeye area [REA], and marbling score [MBS]), directly affecting beef production systems’ profitability. The body protein and fat content, which determine carcass traits, can potentially contribute to the variability in feed efficiency by influencing the energy status [7]. Nkrumah et al [12] reported a low but significant phenotypic correlation between RFI and ultrasound BFT (r = 0.25) and CWT (r = 0.26) and a very low correlation with loin muscle area (LMA; r = 0.09) and MBS (r = 0.03). They also indicated that the rate of increase in BFT was higher in animals with a high RFI (low efficiency). Similarly, Parsons et al [13] reported that animals with a low RFI (high efficiency) had a thinner (0.65 vs 0.76 cm) ultrasound BFT than those with a high RFI (low efficiency), although the LMA did not differ according to RFI. In contrast, Lancaster et al [14] reported little phenotypic correlation of RFI with both LMA and BFT. This tendency was observed for both RFI and FCR. Santana et al [15] reported a low correlation (r<0.2) between FCR and carcass traits (i.e., BFT, REA, and rump fat thickness) measured using ultrasound. Torres-Vázquez et al [16] reported considerably low genetic and phenotypic correlations (r<0.3) between the FCR and carcass traits, including REA, intramuscular fat, and CWT. Furthermore, the correlation between carcass traits and feed efficiency may differ by breed [17]. Hanwoo is known for high intramuscular fat deposition, and thus, there is a strong rationale to examine the association between carcass traits and feed efficiency over the entire feeding period in Hanwoo steers, which has not been tested.

Therefore, the objectives of this study were: i) to investigate whether the two indices for feed efficiency in beef cattle, RFI and FCR, could be repeated throughout the entire feeding stage, and ii) to determine whether feed efficiency is related to carcass traits after slaughter in Hanwoo steers.

## MATERIALS AND METHODS

This study was conducted at the Center for Animal Science Research, Chungnam National University, Republic of Korea. The use of animals and protocols for this experiment were reviewed and pre-approved by the Chungnam National University Animal Research Ethics Committee (CNU-01021).

### Animals, housing, experimental design, and management

Fifty nine-month-old Hanwoo steers were enrolled in the study. All animals of the same age (eight month), purchased from the commercial Hanwoo market (Jeonbuk, Korea), were vaccinated upon arrival at the Chungnam National University Animal Science Research Center and, after a two-week adaptation period, were surgically castrated by a veterinarian. The initial mean BW (±standard deviation [SD]) of the steers was 256 (±22.7) kg. The steers were housed in one of the four pens (12 to 13 steers per each pen). Each pen (10×10 m) was equipped with an automatic concentrate feeding station (ACFS) and four forage feed bunks (Dawoon Co., Incheon, Korea), with sawdust laid on the floor as bedding. Each ACFS (1.9×2×0.8 m) and forage feed bunk automatically measured individual feed intake, recognizing each steer using a radio-frequency identification neck tag attached to each steer. Within each pen, the steers consumed the same diet (i.e., concentrate mix and forage). Throughout the experiment, sawdust bedding in the pen was replaced every month or two, and all steers had free access to drinking water and a mineral block.

**Figure 1:**
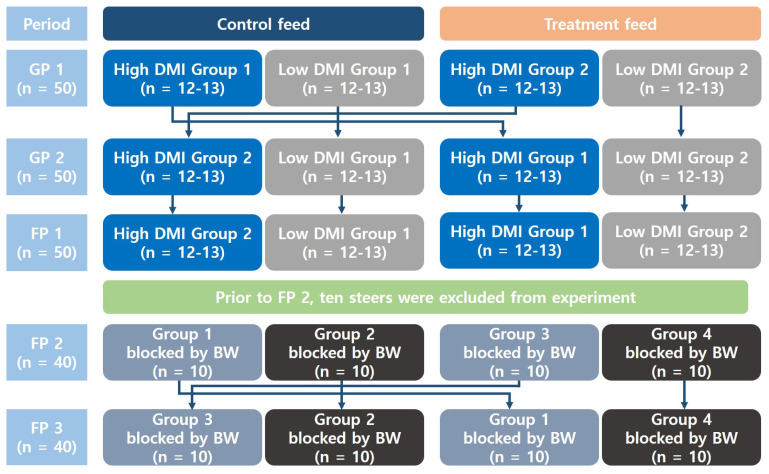
The design in which animals were allocated to control and treatment groups for each period. GP 1, growing period 1; GP 2, growing period 2; FP 1, fattening period 1; FP 2, fattening period 2; FP 3, fattening period 3; DMI, dry matter intake; BW, body weight.

The entire feeding stage was divided into five periods (i.e., two growing periods and three fattening periods) with diet changes. In each period, two different dietary treatments (control vs treatment) were applied using a completely randomized block design [18]. Growing period (GP) 1 lasted for three months, from December 2018 to March 2019, and GP 2 lasted for two months, from March 2019 to May 2019. Fattening period (FP) 1 lasted for six months, from May 2019 to November 2019; FP 2 lasted for three months, from December 2019 to March 2020; and FP 3 lasted for the last six months, from May 2020 to September 2020. Between each period, there was an adaptation period of 1 to 2 weeks to eliminate the effect of the feed treatment. The design in which animals were allocated to control and treatment groups for each period is depicted in Figure 1. One month before the start of GP 1, the steers, stratified based on dry matter intake (DMI) as a percentage of BW in the preceding pilot period, in which all animals were fed the same diet (i.e., concentrate mix and forage) for one month, were randomly allocated into four groups of 12 to 13 steers (two high and two low DMI groups). Each group was housed in a single pen. One high and one low DMI group were randomly paired and assigned to the control group, which was fed the commercial concentrate mix, while the remaining two groups were subjected to the dietary treatment. Prior to the beginning of GP 2, the high DMI group was switched between the control and treatment groups, and the dietary group assignment remained the same until FP 1. Prior to FP 2, ten steers were excluded from the experiment because of abnormally low or high ADG or intake compared with the average. The remaining 40 steers were blocked by BW and randomly allocated to four groups of ten steers. Dietary treatments (control vs treatment) were randomly assigned to the groups during each feeding period. Before FP 3, one control group and one treatment group were switched.

For each period, the control was a commercial concentrate mix (Hanwoo Maru, Woosung Feed Co. Ltd., Daejeon, Korea), and the dietary treatment was a concentrate mix formulated to provide different levels of nutrients (Table 1). Briefly, the dietary treatments (control vs treatment) for GP 1, GP 2, FP 1, FP 2, and FP 3 were crude protein (CP) level (20% vs 22% dry matter [DM]), energy content in terms of total digestible nutrients (TDN; 76% vs 72% DM), TDN-to-CP ratio (72-18 vs 73-17.56), supplementation of corn steep liquor (CSL; 0 vs up to 2%), and starch content (37% vs 39% DM), respectively. The steers were fed the same forage irrespective of dietary treatment during each period (tall fescue during the growing period and annual ryegrass straw during the fattening period). The dietary ingredients and chemical compositions of the concentrate mixes and forages are described in Supplementary Tables S1 to S10. The nutrient composition and amount of concentrate mix provided were determined according to the RDA [19], aiming at an ADG of 0.95, 0.95, 0.95, 0.97, and 0.67 kg in GP 1, GP 2, FP 1, FP 2, and FP 3, respectively.

During the entire feeding stage, forage was fed *ad libitum* twice daily, at 08:00 and 18:00 h, and a concentrate mix was provided through the ACFS. The gate of the ACFS remains open when animals are absent. However, once an RFID neck tag is detected by the feeder for 2 s, the system identifies the steer’s presence, causing the ACFS gate to close, allowing only one steer at a time. The door reopens if the designated amount of concentrate mix is entirely consumed or if the RFID neck tag is not sensed for more than 70 s, regardless of the steer’s presence inside the station. The feeder dispenses a pre-set amount of concentrate mix to each steer based on its daily allowance and meal frequency. Within a given time frame, the feeder allocates the mix according to a fixed size (80 to 90 g) in the ACFS. For example, when a steer is allowed to consume 2 kg during one time frame, with an 80 g fixed size, the feeder would dispense the mix 25 times in one time frame. The duration of each time frame is calculated by dividing 24 hours by the number of meals (i.e., meal frequency), the daily feed allowance is uniformly spread across these periods. Any unconsumed portion from one time frame can be eaten in the subsequent one. The forage feed bunk deployed incorporated dual load cells for tracking leftover feed, sensors that recognize the presence of cattle through RFID ear tags, and a panel that moves up or down depending on whether a steer is detected. If a steer does not approach the forage feed bunk, or if it does but its RFID tag is not detected by the sensor, the panel remains raised, preventing the steer from consuming forage. Once a steer’s RFID tag is recognized by the sensor, the panel lowers, allowing the steer to access the forage. With the panel lowered, only one steer at a time can consume forage. The forage intake was recorded as the difference in weight before entry and after exit. Daily concentrate mix allowance were as follows: GP 1 (4.2 kg), GP 2 (5.5 kg), FP 1 (8.3 kg), FP 2 (9.8 kg), and FP3 (9.8 kg). During the growing periods (i.e., GP 1 and GP 2), each day was divided into four-time frames of 6 h, and the steers were able to consume up to one-fourth of the daily allowable concentrate mix (kg/d) during each frame. During the fattening periods (i.e., FP 1, FP 2, and FP 3), each day was divided into six time frames of 4 h each, and the steers were able to consume up to one-sixth of the daily allowable concentrate mix (kg/d).

### Measurements and analyses

The BW of the steers was measured every four weeks before morning feeding during the entire experimental period. Every four weeks, each steer’s recorded daily feed intakes were processed. Daily intakes of > or <2.5 times the SD from the mean intake over four weeks were treated as outliers, and a total of 1.8% of intake data collected during the experiment was removed. The intake on the day when management could affect feed intake (e.g., bedding replacement, BW measurement, and foot-and-mouth disease vaccination) was also removed. All steers were slaughtered between September 2020 and March 2021, after reaching a target live BW of 800 kg. Steers that reached the target BW were transported to a commercial slaughterhouse (Nonghyup Livestock Joint Market Eumseong Center, Chungcheongbuk-do, Korea). At the slaughterhouse, steers were fasted for 24 h and weighed before slaughter. Carcass characteristics (i.e., CWT, BFT, ribeye area, marbling score) were evaluated using the Korean Grading System for Livestock Products (MAFRA, 2018).

Diets for each feeding stage were sampled once every four weeks. The feed samples were dried at 60°C for 96 h and ground through a cyclone mill (Foss, Hillerød, Denmark) fitted with a 1 mm screen. The nutrient composition of the samples composited for each feeding period was analyzed at Cumberland Valley Analytical Services Inc. (Hagerstown, MD, USA). The details of the methods used to analyze the nutrient content of the samples were the same as those described by Jeon et al [20]

The RFI of each animal at each feeding stage was calculated by subtracting the predicted DMI from the observed DMI. As a result, a smaller RFI indicates a higher feed efficiency. The predicted DMI was calculated based on a linear regression of DMI against ADG and MBW (i.e., BW^0.75^) as proposed by Koch et al [6], using the PROC GLM of SAS (SAS Institute Inc., Cary, NC, USA). The model used to calculate RFI involves the linear regression of DMI on ADG and MBW:


$$\text{y}=\beta_{0}+\beta_{1}(\text{ADG})+\beta_{2}(\text{MBW})$$

where y is the predicted DMI, β_0_ is the regression intercept, β_1_ is the partial regression of the DMI on ADG, and β_2_ is the partial regression of the DMI on MBW.

In addition to the RFI, the FCR, calculated by dividing DMI by ADG, of each steer was estimated for each feeding period.

### Statistical analysis

Among the 40 steers that remained in the experiment throughout the feeding stage, four animals that showed disease or feeder adaptation problems were excluded from the data analysis.

No dietary treatments affected feed efficiency except for FP 2, in which the treatment group that was supplemented with CSL up to 2% had a lower RFI (0.20 vs −0.18; p = 0.033). The feeding efficiency indices (RFI and FCR) in each feed period followed a normal distribution; therefore, they were calculated for all steers (n = 36) in each feeding period. Body weight, ADG, and feed intake of the steers during each feeding period are shown in Table 2.

To evaluate the repeatability of feed efficiency, correlations between the rankings of steers according to the feed efficiency index in GP 1 and those in the other periods were estimated. Additionally, repeatability of ADG, DMI, RFI, and FCR over the entire period was analyzed using PROC MIXED of SAS, with animals as a random effect, while period as a fixed effect, according to the following equation [21]:


$$Repeatability=\frac{Animal\;variance}{Animal\;variance+Residual\;variance}$$

According to feed efficiency ranking, steers were classified into three classes (low, medium, and high) with 12 animals. The proportion of steers that remained in the same class as those in GP 1 was examined for each feeding period, and its significance was tested using the chi-square test with the FREQ procedure in SAS (SAS Institute Inc., Cary, NC, USA). The Theil index (H), a normalized entropy-based segregation measure, was also calculated to quantify the consistency of maintaining the class in GP 1 for the rest of the feeding periods, using the SEGREGATION package in R [22].

The association of carcass traits with the RFI and FCR of steers at each feeding stage was also determined by calculating Pearson correlation coefficients.

## RESULTS

### Repeatability of feed efficiency over the entire feeding stage

Significant correlations were observed between the ranking of steers according to RFI during GP 1 and those in the remaining periods (Table 3). The correlation of RFI rankings with GP 1 was the highest in GP 2 (r = 0.65; p<0.001) and remained significant in a regressive manner until FP 2 (r = 0.38; p<0.05). The RFI rankings in GP 1 were highly correlated with those in the overall feeding stage (r = 0.65; p<0.001).

The ranking of steers according to RFI was also significantly correlated with feeding periods. Residual feed intake rankings between the two consecutive periods were significantly correlated (p<0.05; Table 3). The correlation coefficients were higher than 0.6 within GP or FP (p<0.001), while that between GP 2 and FP 1—the transition from GP to FP—was the lowest at 0.4 (p<0.05). Nevertheless, a strong correlation was observed between the overall GPs and FPs (r = 0.52; p<0.01). Fattening period 1 and FP 3, which had the most prolonged experimental period (six months), showed the highest correlation with the overall feeding stage (r = 0.81 and 0.79, respectively) in the rankings of steers according to the RFI.

No significant correlation between GP 1 and the other periods was observed in the ranking of steers based on FCR (Table 4). A significant correlation between the feeding periods was observed only between FP 1 and FP 3 (r = 0.36; p<0.05). The correlation between overall GPs and FPs was negligible (r = 0.16; p>0.05). However, within GP or FP, the steers’ FCR rankings were highly correlated with those during the overall period. Specifically, the correlation coefficients of GP 1 and GP 2 with the overall GP were 0.84 and 0.57, respectively (p<0.001). The correlation coefficients of fattening periods with the overall FP ranged from 0.59 to 0.74 (p<0.001). The steers’ rankings according to FCR during the overall feeding stage were highly correlated with those in the fattening periods (r = 0.62 to 0.67; p<0.001). Consequently, a high correlation (r = 0.94; p<0.001) was observed between the overall FP and the entire feeding stage in the ranking of steers according to FCR.

**Figure 2:**
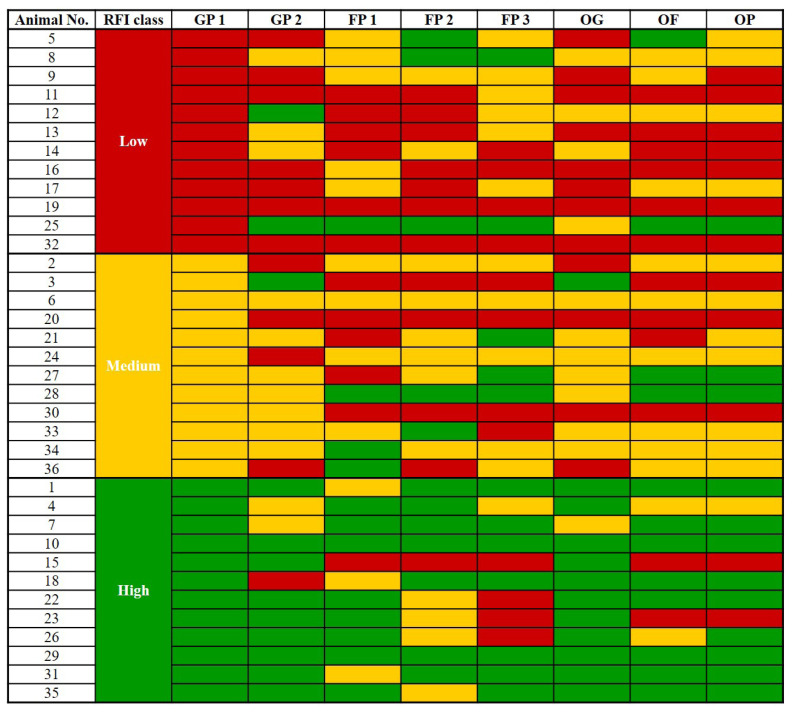
Residual feed intake (RFI) ranking and class in each growing and fattening period of the steers classified as the low (red), middle (yellow), and high (green) RFI class at growing period 1. GP 1, growing period 1; GP 2, growing period 2; FP 1, fattening period 1; FP 2, fattening period 2; FP 3, fattening period 3; OG, overall growing period; OF, overall fattening period; OP, overall feeding period.

Throughout the feeding stages, steers remained in the same feed efficiency classes (i.e., high, medium, and low) based on RFI during GP 1, except for FP 3 (p<0.05; Table 5; Figure 2). The high feed-efficiency animals in GP 1 based on RFI comprised 75%, 67%, and 58% of the high class in GP 2, FP 1, and FP 2, respectively (p<0.05; Table 5). Overall, 75%, 58%, and 58% of steers classified as having high, medium, and low feed efficiency, respectively, based on RFI during GP 1 remained in the same feed-efficient class. Interestingly, the tendency to maintain the feed efficiency class was greater in highly efficient steers, with 75%, 67%, 58%, and 75% of steers ranked in the high class during GP 1 remaining in the high class during GP 2, FP 1, FP 2, and the overall period, respectively.

**Figure 3:**
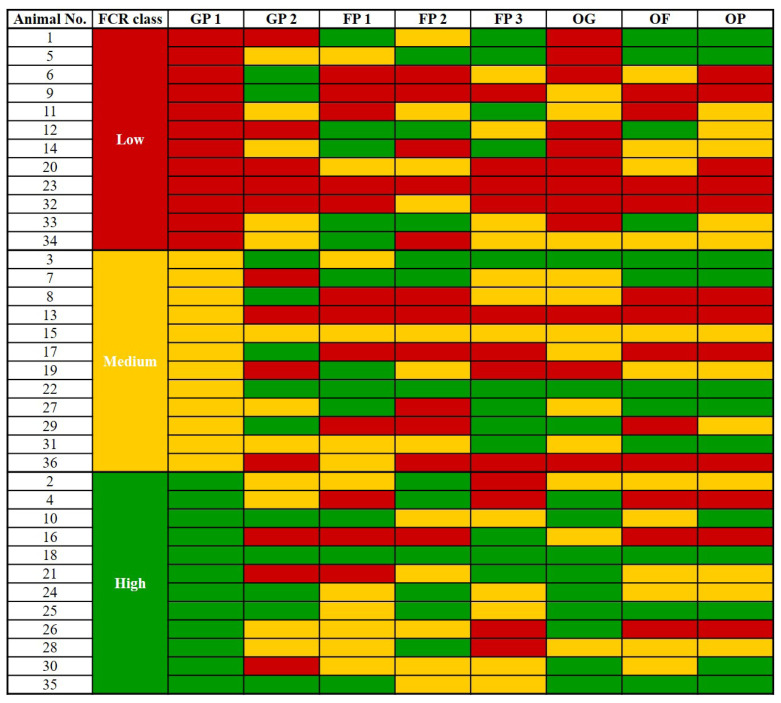
Feed conversion ratio (FCR) ranking and class in each growing and fattening period of the steers classified as the low (red), middle (yellow), and high (green) FCR class at growing period 1. GP 1, growing period 1; GP 2, growing period 2; FP 1, fattening period 1; FP 2, fattening period 2; FP 3, fattening period 3; OG, overall growing period; OF, overall fattening period; OP, overall feeding period.

In contrast, the feed efficiency classes during GP 1 based on FCR were unrelated to those during the other feeding periods (p>0.05; Table 5; Figure 3). Regardless of the feed efficiency class during GP 1 based on FCR, steers were randomly distributed into three feed-efficiency classes in subsequent feeding periods. For instance, among the steers classified as medium during GP 2, four, three, and five were classified as high, medium, and low during GP 1, respectively. Overall, based on the FCR, <50% of the steers remained in the same feed efficiency classes as during GP 1.

**Figure 4:**
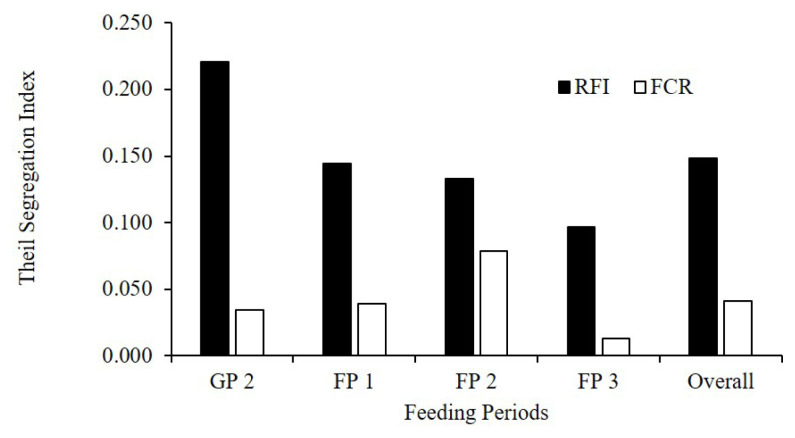
Theil segregation index of the feed efficiency classes in feeding periods based on the feed efficiency indexes. The steers classified as high, middle, and low efficient groups, 12 steers each, based on residual feed intake (RFI; black bar) or feed conversion ratio (FCR; white bar). GP is growing period, and FP is fattening period.

The Theil segregation index also indicated a tendency to maintain the feed efficiency class of steers according to RFI rather than FCR (Figure 4). The overall Theil index of RFI was 0.15, decreasing from 0.22 for GP 2 to 0.10 for FP 3. The Theil indices of FCR were <0.08 in all feed periods, and the overall index was 0.04. The repeatability calculated using animal variance and residual variance over the entire period was also higher for RFI (0.61) compared with FCR (0.15). The repeatability of ADG was 0.75 while that of concentrate, forage and total DMI were 0.38, 0.60, and 0.58, respectively.

### Association between feed efficiency and carcass traits

Regardless of the feed efficiency indices (RFI and FCR), the correlations between feed efficiency during each feeding period and post-slaughter carcass traits were generally low. No significant correlations were observed between RFI during any feeding period and the evaluated carcass traits (Table 6). However, FCR was significantly correlated with CWT (Table 7). Carcass weight was negatively correlated with FCR during FP 3 (r = −0.400; p<0.05) and the overall fattening period (r = −0.447; p<0.01) and positively correlated with FCR during the entire feeding stage (r = 0.398; p<0.05). Backfat thickness, REA, and MBS were negatively correlated with FCR; however, these correlations were not statistically significant.

## DISCUSSION

The feed efficiency of animals is closely associated with farm profitability, and its consistency or repeatability is of significant interest in selecting efficient animals and determining feeding management plans. Feed efficiency may vary depending on the diet provided to the animals [23]; however, most studies on the repeatability of feed efficiency have evaluated it between two short feeding periods with the same or different diets. This study aimed to examine the repeatability of feed efficiency over an entire feeding period consisting of two GPs and three FPs with diets varying according to the physiological status of the animals.

Our findings indicate that the feed efficiency of steers are consistent between feeding periods, particularly when calculated based on RFI, in accordance with previous research. For example, Kelly et al [8] and Durunna et al [24] reported higher RFI correlations (r = 0.62 and 0.52, respectively) than G:F correlations (r = 0.37 and 0.37, respectively) when replacement heifers were fed the same diet across two periods. Cassady et al [11] found significant correlations in both RFI and FCR between two consecutive feeding periods with different grain-based diets, with the RFI correlation between the two periods being higher (r = 0.63) than that of the FCR (r = 0.41). Durunna et al [10] found significant correlations in both RFI and G:F between two consecutive periods when steers were fed the growing diet (r = 0.44 vs 0.38, respectively), finishing diet (r = 0.42 vs 0.29, respectively), or transitioned from growing to finishing diets (r = 0.33 vs 0.20, respectively). Furthermore, Lahart et al [25] reported that, despite the interval between feeding periods and varying diets, the mean RFI correlation was higher (r = 0.22) than that of G:F (r = 0.02) in steers and heifers. In line with prior studies, our study suggests that RFI is repeatable over the entire feeding period regardless of diet changes and that RFI and FCR are not interchangeable when assessing feed efficiency over the same period.

Residual feed intake is independently associated with ADG and BW [26], whereas FCR influences mature BW and feed intake because of its negative genetic correlation with ADG and BW [5]. Another difference between RFI and FCR is that RFI measures feed efficiency as the difference between actual and predicted DMI, calculated through a linear regression of DMI for ADG and MBW (BW^0.75^) in a herd. In contrast, the FCR calculates feed efficiency as the ratio of ADG to DMI for each individual. Durunna et al [10] argued that owing to these differences, efficient animals vary based on feed efficiency measures, necessitating consideration. Consequently, efficient animals may differ depending on their diet and physiological stage, as determined by the feed efficiency measurement method. Furthermore, the higher repeatability of RFI compared with FCR during the entire feeding period implies that RFI may be a more suitable index for assessing animal feed efficiency.

Residual feed intake showed high repeatability compared with FCR, but we found that the correlation between RFI rankings in each period decreased as the interval between periods increased. Feed efficiency is influenced by various factors, such as diet, metabolic differences [10], rumen microbial populations [27], and feeding behavior [28]. As a result, RFI repeatability can be affected by several factors, similar to other feed efficiency measures. Our study indicates that the interval between the measurement periods may be one of the main factors affecting RFI repeatability. This is consistent with the study by Lahart et al [25] who reported a low correlation between RFI for two periods and attributed this to the long interval between periods. Our findings also suggest that feed efficiency repeatability may decrease during the transition period (i.e., from growing to fattening) due to alterations in nutritional composition, animal physiological state, and maturity level. The correlation between GP 2 and FP 1, the transition from GP to FP, was lower (r = 0.4; p<0.05) than that of the other two consecutive periods (r>0.5; p<0.01). Nevertheless, the correlation between RFI for the overall GP and FP was moderate (r = 0.52), and the correlation between the RFI of the overall GP and FP and the RFI of the overall feeding stage demonstrated a high correlation (r = 0.71 and 0.93, respectively). As a result, although RFI may be affected by the interval between measurement periods (i.e., animal growth), the effect of the measurement interval is weakened, and the RFI of the overall feeding stages can be predicted through RFI during periods with different diets and measurement periods.

Both RFI and FCR showed high correlations (r>0.5; p< 0.001) between the feed efficiency of the GP and FP stages and that of each period corresponding to each stage. These results are consistent with those of previous studies [10,11,24] which reported high correlations (r>0.5) between RFI and G:F of the entire period in each stage and RFI and G:F of each period, even when the entire period in the stage was divided into several periods. These consistent findings, regardless of diet, breed, feed efficiency measurement method, or measurement period, suggest that the feed efficiency of a specific period during the growing or fattening stages can predict the feed efficiency of the entire growing or fattening stage.

Our study found that the correlation between RFI for each period in the fattening stage and RFI for overall feeding stages was higher (r = 0.75 to 0.81; p<0.001) than the correlation between RFI for each period in the growing stage and RFI for overall feeding stages (r = 0.58 to 0.65; p<0.001). Similarly, the correlation between the FCR for each period in the fattening stage and the overall feeding stages (r = 0.62 to 0.67; p<0.001) was higher than that of the growing stage (r = 0.32; p>0.05). These findings imply that regardless of the repeatability or measurement method of feed efficiency, it may be more appropriate to predict the overall feed efficiency of animals using measurements from the FP rather than the GP. Durunna et al [10] stated that early recognition of efficient animals is crucial for the genetic enhancement of feed efficiency, but it is more appropriate to measure feed efficiency closer to the animal’s mature BW. These findings have also been reported in heifers. Durunna et al [24] measured feed efficiency and found a strong correlation with feed efficiency during the entire measurement period. However, previous studies reported contradictory results. For example, Cassady et al [11] reported a higher correlation between the first measured feed efficiency and feed efficiency for the entire period when steers and heifers were fed the same feed for two periods. The difference in this result might be due to the fact that Cassady et al [11] provided the same feed for both periods, and the age of the animals in the later measured period still corresponded to the growing stage in our study.

In this study, ADG exhibited a high repeatability (0.75) throughout the entire period, and DMI also demonstrated a moderate repeatability (0.58). These results contradict previous research findings that reported low repeatability of ADG along with moderate repeatability of DMI [8,11]. The difference in these results could stem from variations in the periods over which repeatability was measured. Cassady et al [11] suggested that despite the high repeatability of RFI, the low repeatability of ADG could be attributed to the short measurement period, because even though DMI explains the majority of the RFI variability, ADG also contributes to a portion of the RFI variability. Moreover, if compensatory growth in animals occurs when the period for measuring repeatability is short, there is a higher likelihood that the repeatability of ADG over time will be even lower. Therefore, our results suggest that over the entire period, rather than in two specific periods, ADG exhibits higher repeatability than DMI. Additionally, individuals that exhibit good growth during the GP may maintain a similar growth rate during the FP.

Among the three RFI classes in GP 1, the proportion of high class animals maintaining their class over time was higher than that of the low and medium classes. Additionally, the proportion of animals that changed two classes (i.e., from low to high, or vice versa) in the RFI class of GP 1 was smaller than that of those that changed one class. These results indicate that it is difficult for efficient or inefficient animals in the GP to change their feed efficiency in the opposite direction and suggest that animals that are inefficient during the GP have a high probability of remaining inefficient throughout their lifetime. Furthermore, this finding implies that even if feed efficiency at the fattening stage is suitable for predicting overall feed efficiency, identifying inefficient animals is possible even during the GP. Similar results have been reported in previous studies. In beef steers, when the same diet was provided or the diet was changed for two consecutive periods, the proportion of change from RFI high class to low class was approximately 10% higher than the opposite change [10]. Durunna et al [24] found that the proportion of heifers maintaining the RFI high class for two periods was higher than that of heifers retaining the RFI low class, and the proportion of class change from low to high or vice versa was approximately 6%. These results were similar, even when there was an interval between the feed efficiency measurement periods. The proportion of change in two RFI classes during the two periods in heifers and steers was approximately 30% higher than the rate at which one RFI class changed [25].

In our study, there was a weak correlation (r<0.5) between feed efficiency and carcass traits, regardless of the period or method of feed efficiency measurement. Similarly, previous studies [29–33] reported a low correlation between feed efficiency and carcass traits. Regardless of breed, these consistent results suggest that even if the genetic correlation between feed efficiency and carcass traits is high, feed efficiency calculated using only growth rate and live BW cannot explain carcass traits such as body fat deposition and CWT. Kelly et al [34] indicated that MBW (BW^0.75^) should be replaced with CWT in RFI calculations because the rate of change in fat, bone, and muscle, the morphological difference in the size of internal organs, and the ability to convert energy into an increase in BW do not necessarily lead to an increase in CWT, resulting in an inequality between an increase in BW and an increase in CWT. Santana et al [31] argued that for efficient production, when selecting individuals in a beef cattle production system, an animal should be efficient not only for feed efficiency, but also for the disposal of body fat. As a result, although RFI in this study was calculated using only MBW and ADG as variables, further studies should consider determining the association between carcass traits and RFI by calculating RFI with carcass traits as variables.

## CONCLUSION

We found that RFI demonstrated moderate repeatability throughout the entire feeding stage, regardless of diet, and exhibited higher repeatability than FCR. However, repeatability decreased as the feeding period progressed. As in previous studies, the strong correlation between the feed efficiency of a specific period and the corresponding stage suggests that it is unnecessary to measure feed efficiency for all periods within a given stage (i.e., growing or fattening stage). Our findings indicate that evaluating feed efficiency during the fattening stage may be more appropriate than using measurements from the growing stage to assess feed efficiency during the overall feeding period. Nonetheless, relying solely on feed efficiency measurements during the GP could lead to the selection of inefficient animals, as these animals are more likely to continue to exhibit inefficiency during the fattening stage than their efficient counterparts. There was a low correlation between feed efficiency and carcass traits, indicating that feed efficiency calculated using only growth rate and live BW cannot explain carcass traits such as body fat deposition and CWT.

## Figures and Tables

**Table 1:** Design of dietary treatments of the entire experimental period

Stage	Period (n, mo)^1)^	Concentrate mix^2)^		Forage^3)^

		Control	Treatment	
Growing	GP 1 (50, 3 mo)	CP, 20% DM	CP, 22% DM	Tall fescue
	GP 2 (50, 2 mo)	TDN, 76% DM	TDN, 72% DM	Tall fescue
Fattening	FP 1 (50, 6 mo)	TDN-to-CP ratio, 72-18	TDN-to-CP ratio, 73-17.56	Annual ryegrass
	FP 2 (40, 3 mo)	CSL, 0%	CSL, gradually up to 2%	Annual ryegrass
	FP 3 (40, 6 mo)	Starch, 37% DM	Starch, 39% DM	Annual ryegrass

1) GP, growing period; FP, fattening period.

2) CP, crude protein; DM, dry matter; TDN, total digestible nutrients; CSL, supplementation of corn steep liquor in concentrate mix.

3) Steers were fed the same forage irrespective of dietary treatment in each period.

**Table 2:** Body weight, average daily gain, feed intake in each feeding period

Items^1)^	Feeding period^2)^															

	GP 1		GP 2		FP 1		FP 2		FP 3		Overall GP		Overall FP		Overall	
							
	Mean	SD	Mean	SD	Mean	SD	Mean	SD	Mean	SD	Mean	SD	Mean	SD	Mean	SD
Initial BW (kg)	259	19.7	348	21.7	399	21.3	585	30.0	666	39.4	259	19.7	399	21.3	259	19.7
Final BW (kg)	348	21.7	399	21.3	553	26.4	666	39.4	760	55.1	399	21.3	760	55.1	760	55.1
ADG (g/d)	991	127.0	757	92.8	838	90.0	893	153.8	519	138.0	891	80.8	746	90.9	782	71.1
DMI (kg/d)																
Concentrate	3.8	0.11	5.1	0.05	7.5	0.17	8.5	0.45	7.5	0.60	4.3	0.07	7.7	0.34	6.8	0.26
Forage	3.0	0.34	3.3	0.42	2.1	0.33	1.2	0.29	1.5	0.32	3.1	0.35	1.7	0.29	2.0	0.26
Total	6.8	0.42	8.4	0.43	9.6	0.40	9.7	0.59	9.0	0.73	7.4	0.39	9.4	0.51	8.9	0.44
Feed efficiency																
RFI	0.0	0.42	0.0	0.41	0.0	0.39	0.0	0.52	0.0	0.58	0.0	0.39	0.0	0.43	0.0	0.39
FCR	6.9	1.04	11.3	1.70	11.5	1.29	11.1	1.66	18.6	5.34	8.4	0.89	12.7	1.32	11.4	0.98

1) BW, body weight; ADG, average daily gain; DMI, dry matter intake; RFI, residual feed intake; FCR, feed conversion ratio, DMI (g)/ADG (g).

2) GP, growing period; FP, fattening period; SD, standard deviation.

**Table 3:** Spearman correlation coefficients of residual feed intake between feeding periods

Period^1)^	GP 1	GP 2	FP 1	FP 2	FP 3	Overall GP	Overall FP	Overall
GP 1	1.00	0.65***	0.44**	0.38*	0.32	0.90***	0.45**	0.65***
GP 2	-	1.00	0.40*	0.36*	0.27	0.89***	0.41*	0.58***
FP 1	-	-	1.00	0.59***	0.50**	0.51**	0.77***	0.81***
FP 2	-	-	-	1.00	0.62***	0.44**	0.77***	0.75***
FP 3	-	-	-	-	1.00	0.35*	0.86***	0.79***
Overall GP	-	-	-	-	-	1.00	0.52**	0.71***
Overall FP	-	-	-	-	-	-	1.00	0.93***
Overall	-	-	-	-	-	-	-	1.00

1) GP, Growing period; FP, Fattening period.

* p<0.05;

** p<0.01;

*** p<0.001.

**Table 4:** Spearman correlation coefficients of feed conversion ratio between feeding periods

Period^1)^	GP 1	GP 2	FP 1	FP 2	FP 3	Overall GP	Overall FP	Overall
GP 1	1.00	0.10	−0.01	0.32	−0.06	0.84***	0.07	0.32
GP 2	-	1.00	0.02	0.10	0.23	0.57***	0.20	0.32
FP 1	-	-	1.00	0.24	0.36*	−0.02	0.74***	0.67***
FP 2	-	-	-	1.00	0.15	0.31	0.59***	0.62***
FP 3	-	-	-	-	1.00	0.11	0.70***	0.67***
Overall GP	-	-	-	-	-	1.00	0.16	0.43*
Overall FP	-	-	-	-	-	-	1.00	0.94***
Overall	-	-	-	-	-	-	-	1.00

1) GP, growing period; FP, fattening period.

* p<0.05;

** p<0.01;

*** p<0.001.

**Table 5:** Proportion of the steers that maintained the same feed efficiency class of growing period 1 in each feeding period

Feed efficiency class^1)^	Feeding period^2)^																				

	GP 2			FP 1			FP 2			FP 3			Overall GP			Overall FP			Overall		
						
	n	%	p-value	n	%	p-value	n	%	p-value	n	%	p-value	n	%	p-value	n	%	p-value	n	%	p-value
Based on RFI																					
High	9	75	0.002	8	67	0.033	7	58	0.040	7	58	0.136	11	92	<0.001	8	67	0.040	9	75	0.002
Medium	7	58		4	33		6	50		5	42		7	58		6	50		7	58	
Low	7	58		6	50		7	58		4	33		8	67		5	50		7	58	
Based on FCR																					
High	5	42	0.645	3	25	0.558	6	50	0.240	3	25	0.910	9	75	<0.001	3	25	0.558	5	42	0.645
Medium	3	25		4	33		3	25		3	25		6	50		2	17		3	25	
Low	5	42		5	42		5	42		4	33		9	75		4	33		5	42	

1) RFI, residual feed intake; FCR, feed conversion ratio.

2) GP, Growing period; FP, Fattening period.

**Table 6:** Pearson correlation between residual feed intake of each feeding stage and carcass traits that after slaughter

Period^1)^	Carcass traits^2)^			

	CWT	BFT	REA	MBS
GP 1	0.167	−0.156	0.222	0.256
GP 2	0.301	−0.222	0.164	0.097
FP 1	0.114	−0.095	0.070	−0.084
FP 2	0.002	0.147	−0.057	−0.127
FP 3	0.043	−0.081	−0.104	−0.109
Overall GP	0.212	−0.198	0.195	0.200
Overall FP	−0.003	−0.078	−0.017	−0.125
Overall	0.091	−0.099	0.051	−0.034

1) GP, growing period; FP, fattening period.

2) CWT, carcass weight, kg; BFT, backfat thickness, mm; REA, ribeye area, cm^2^; MBS, marbling score, unitless.

* All correlations were not significant (p>0.05).

**Table 7:** Pearson correlation between feed conversion ratio of each feeding stage and carcass traits that after slaughter

Period^1)^	Carcass traits^2)^			

	CWT	BFT	REA	MBS
GP 1	0.103	0.118	0.005	0.012
GP 2	−0.252	0.033	−0.341	−0.207
FP 1	−0.274	−0.346	0.160	−0.054
FP 2	−0.329	−0.168	−0.232	−0.184
FP 3	−0.400*	−0.098	−0.167	0.008
Overall GP	−0.012	0.088	−0.133	−0.108
Overall FP	−0.447**	−0.235	−0.142	−0.013
Overall	0.398*	−0.165	−0.184	−0.074

1) GP, growing period; FP, fattening period.

2) CWT, carcass weight, kg; BFT, backfat thickness, mm; REA, ribeye area, cm^2^; MBS, marbling score, unitless.

* p<0.05;

** p<0.01.

## SUPPLEMENTARY MATERIAL

**Supplementary Table S1**. Diet composition (g/kg DM or as stated) of the concentrate mixes in growing period 1

**Supplementary Table S2**. Analyzed chemical composition (g/kg DM or as stated) of the feeds in growing period 1

**Supplementary Table S3**. Diet composition (g/kg DM or as stated) of the concentrate mixes in growing period 2

**Supplementary Table S4**. Analyzed chemical composition (g/kg DM or as stated) of the feeds in growing period 2

**Supplementary Table S5**. Diet composition (g/kg DM or as stated) of the concentrate mixes in fattening period 1

**Supplementary Table S6**. Analyzed chemical composition (g/kg DM or as stated) of the feeds in fattening period 1

**Supplementary Table S7**. Diet composition (g/kg DM or as stated) of the concentrate mixes in fattening period 2

**Supplementary Table S8**. chemical composition (g/kg DM or as stated) of the feeds in fattening period 2

**Supplementary Table S9**. Diet composition (g/kg DM or as stated) of the concentrate mixes in fattening period 3

**Supplementary Table S10**. Analyzed chemical composition (g/kg DM or as stated) of the feeds in fattening period 3
