# Supplementary material for: Repeatability of feed efficiency and its relationship with carcass traits in Hanwoo steers during their entire growing and fattening period
Source: Anim Biosci. 2024 Apr 25;37(9):1568–80. doi: 10.5713/ab.24.0074 (PMC11366531; doi:10.5713/ab.24.0074)
Supplement: Supplementary file 1 [file ab-24-0074-Supplementary-Table-1.pdf]

Supplementary Table 1. Diet composition (g/kg DM or as stated) of the concentrate mixes in growing period 1.

	Treatment[1]	
Items[2]	Commercial	High CP
Corn, flaked	192	193
Wheat, ground	99	157
Corn, ground	8	7
Lupin, flaked	31	31
Coconut oil meal	56	57
DDGS	0	121
Soybean meal	96	96
Rapeseed meal	30	28
Palm kernel meal	71	57
Corn gluten feed	164	82
Wheat bran	118	69
Beet pulp pellet	20	20
Rice bran	21	0
Cottonseed hull	9	0
Limestone	34	34
Molasses	22	22
CMS	11	11
Salt	8	8
Sodium bicarbonate	6	6
Vitamin and mineral mix[3]	3	3

[1]CP, Crude protein

[2]DDGS, Distillers dried grains; CMS, Condensed molasses solubles.

[3]33,330,000 IU/kg vitamin A, 40,000,000 IU/kg vitamin D, 20.86 IU/kg vitamin E, 20 mg/kg Cu, 90 mg/kg Mn, 100 mg/kg Zn, 250 mg/kg Fe, 0.4 mg/kg I, and 0.4 mg/kg Se.
