# Supplementary material for: Repeatability of feed efficiency and its relationship with carcass traits in Hanwoo steers during their entire growing and fattening period
Source: Anim Biosci. 2024 Apr 25;37(9):1568–80. doi: 10.5713/ab.24.0074 (PMC11366531; doi:10.5713/ab.24.0074)
Supplement: Supplementary file 2 [file ab-24-0074-Supplementary-Table-2.pdf]

Supplementary Table 2. Analyzed chemical composition (g/kg DM or as stated) of the feeds in growing period 1

	Treatment[1]		
Items[2]	Commercial	High CP	Tall fescue
DM, g/kg as fed	870	868	957
OM	934	913	946
CP	204	220	57
SOLP	60	69	22
NDICP	25	23	15
ADICP	11	12	10
aNDF	275	255	701
ADF	132	126	464
ADL	33	31	63
Ether extract	43	40	13
Ash	66	87	54
Ca	19	16	3
P	6	6	1
K	12	12	17
Na	5	5	2
Cl	7	8	6
S	4	4	1
Mg	5	4	1
TDN	759	743	552
NEm, MJ/kg DM	7.5	7.3	5.2
NEg, MJ/kg DM	4.9	4.7	2.8
Total carbohydrates	687	654	876
NFC	437	423	191
Carbohydrate fraction, g/kg carbohydrate			
CA	98	86	75
CB1	394	443	7
CB2	144	116	135
CB3	250	242	610
CC	114	113	173
Protein fraction, g/kg CP			
PA+B1	294	312	386
PB2	583	584	349
PB3	67	51	95
PC	56	53	170

[1]CP, Crude protein

[2]DM: dry matter, OM: organic matter, CP: crude protein, SOLP: soluble CP, NDICP: neutral detergent insoluble CP, ADICP: acid detergent insoluble CP, aNDF: neutral detergent fiber analyzed using a heat stable amylase and expressed inclusive of residual ash, ADF: acid detergent fiber, ADL: acid detergent lignin, TDN: total digestible nutrients, NEm: net energy for maintenance, NEg: net energy for growth, NFC: non-fiber carbohydrate, CA: carbohydrate A fraction; ethanol soluble carbohydrates, CB1: carbohydrate B1 fraction; starch, CB2: carbohydrate B2 fraction; soluble fiber, CB3: carbohydrate B3 fraction; available insoluble fiber, CC: carbohydrate C fraction; unavailable carbohydrate, PA+B1: protein A and B1 fractions; soluble CP, PB2: protein B2 fraction; intermediate degradable CP, PB3: protein B3 fraction; slowly degradable fiber-bound CP, PC: protein C fraction; unavailable CP.
