# Supplementary material for: Repeatability of feed efficiency and its relationship with carcass traits in Hanwoo steers during their entire growing and fattening period
Source: Anim Biosci. 2024 Apr 25;37(9):1568–80. doi: 10.5713/ab.24.0074 (PMC11366531; doi:10.5713/ab.24.0074)
Supplement: Supplementary file 3 [file ab-24-0074-Supplementary-Table-3.pdf]

Supplementary Table 3. Diet composition (g/kg DM or as stated) of the concentrate mixes in growing period 2

	Treatment	
Items[1]	Commercial	Low energy
Corn, flaked	200	200
Wheat, ground	60	70
Alfalfa pellet	0	20
Lupin, flaked	30	40
DDGS	100	160
Soybean meal	45	34
Rapeseed meal	30	30
Palm kernel meal	90	90
Wheat flour	30	35
Corn gluten feed	160	170
Wheat bran	149	39
Beet pulp pellet	20	20
Limestone	30	30
Molasses	20	30
CMS	15	15
Salt	7	7
Sodium bicarbonate	3	2
Magnesium oxide	2	1
Vitamin and mineral mix[2]	9	7

[1]DDGS, Distillers dried grains; CMS, Condensed molasses solubles.

[2]33,330,000 IU/kg vitamin A, 40,000,000 IU/kg vitamin D, 20.86 IU/kg vitamin E, 20 mg/kg Cu, 90 mg/kg Mn, 100 mg/kg Zn, 250 mg/kg Fe, 0.4 mg/kg I, and 0.4 mg/kg Se.
