# Supplementary material for: Repeatability of feed efficiency and its relationship with carcass traits in Hanwoo steers during their entire growing and fattening period
Source: Anim Biosci. 2024 Apr 25;37(9):1568–80. doi: 10.5713/ab.24.0074 (PMC11366531; doi:10.5713/ab.24.0074)
Supplement: Supplementary file 4 [file ab-24-0074-Supplementary-Table-4.pdf]

Supplementary Table 4. Analyzed chemical composition (g/kg DM or as stated) of the feeds in growing period 2

Items[1]	Treatment		Tall fescue
	Commercial	Low energy	
DM, g/kg as fed	870	879	957
OM	934	907	946
CP	204	207	57
SOLP	60	83	22
NDICP	25	31	15
ADICP	11	13	10
aNDF	275	320	701
ADF	132	164	464
ADL	33	35	63
Ether extract	43	42	13
Ash	66	93	54
Ca	19	17	3
P	6	6	1
K	12	12	17
Na	5	5	2
Cl	7	7	6
S	4	4	1
Mg	5	4	1
TDN	759	716	552
NEm, MJ/kg DM	7.5	6.9	5.2
NEg, MJ/kg DM	4.9	4.3	2.8
Total carbohydrates	687	658	876
NFC	437	369	191
Carbohydrate fraction, g/kg carbohydrate			
CA	98	103	75
CB1	394	350	7
CB2	144	108	135
CB3	250	312	610
CC	114	128	173
Protein fraction, g/kg CP			
PA+B1	294	401	386
PB2	583	451	349
PB3	67	83	95
PC	56	65	170

[1]DM: dry matter, OM: organic matter, CP: crude protein, SOLP: soluble CP, NDICP: neutral detergent insoluble CP, ADICP: acid detergent insoluble CP, aNDF: neutral detergent fiber analyzed using a heat stable amylase and expressed inclusive of residual ash, ADF: acid detergent fiber, ADL: acid detergent lignin, TDN: total digestible nutrients, NEm: net energy for maintenance, NEg: net energy for growth, NFC: non-fiber carbohydrate, CA: carbohydrate A fraction; ethanol soluble carbohydrates, CB1: carbohydrate B1 fraction; starch, CB2: carbohydrate B2 fraction; soluble fiber, CB3: carbohydrate B3 fraction; available insoluble fiber, CC: carbohydrate C fraction; unavailable carbohydrate, PA+B1: protein A and B1 fractions; soluble CP, PB2: protein B2 fraction; intermediate degradable CP, PB3: protein B3 fraction; slowly degradable fiber-bound CP, PC: protein C fraction; unavailable CP.
