# Supplementary material for: Repeatability of feed efficiency and its relationship with carcass traits in Hanwoo steers during their entire growing and fattening period
Source: Anim Biosci. 2024 Apr 25;37(9):1568–80. doi: 10.5713/ab.24.0074 (PMC11366531; doi:10.5713/ab.24.0074)
Supplement: Supplementary file 6 [file ab-24-0074-Supplementary-Table-6.pdf]

**Supplementary Table 6.** Analyzed chemical composition (g/kg DM or as stated) of the feeds in fattening period 1

| Items[2] | Treatment[1] Commercial | High TDN:CP | Annual ryegrass |
|---|---|---|---|
| DM, g/kg as fed | 875 | 878 | 900 |
| OM | 908 | 912 | 936 |
| CP | 181 | 175 | 56 |
| SOLP | 61 | 58 | 20 |
| NDICP | 32 | 25 | 15 |
| ADICP | 13 | 13 | 12 |
| aNDF | 316 | 301 | 739 |
| ADF | 143 | 149 | 517 |
| ADL | 34 | 35 | 82 |
| Ether extract | 40 | 44 | 8 |
| Ash | 92 | 88 | 64 |
| Ca | 15 | 14 | 5 |
| P | 6 | 6 | 1 |
| K | 11 | 10 | 8 |
| Na | 4 | 4 | 4 |
| Cl | 7 | 8 | 3 |
| S | 3 | 3 | 2 |
| Mg | 4 | 4 | 1 |
| TDN | 718 | 727 | 489 |
| NEm, MJ/kg DM | 7.0 | 7.1 | 3.9 |
| NEg, MJ/kg DM | 4.4 | 4.5 | 1.6 |
| Total carbohydrates | 686 | 692 | 872 |
| NFC | 403 | 416 | 147 |
| Carbohydrate fraction, g/kg carbohydrate | | | |
| CA | 77 | 72 | 42 |
| CB1 | 415 | 449 | 11 |
| CB2 | 94 | 80 | 116 |
| CB3 | 295 | 279 | 604 |
| CC | 118 | 121 | 226 |
| Protein fraction, g/kg CP | | | |
| PA+B1 | 336 | 328 | 359 |
| PB2 | 490 | 529 | 369 |
| PB3 | 100 | 66 | 52 |
| PC | 74 | 76 | 221 |

[1]TDN, Total digestible nutrients; CP, Crude protein

[2]DM: dry matter, OM: organic matter, CP: crude protein, SOLP: soluble CP, NDICP: neutral detergent insoluble CP, ADICP: acid detergent insoluble CP, aNDF: neutral detergent fiber analyzed using a heat stable amylase and expressed inclusive of residual ash, ADF: acid detergent fiber, ADL: acid detergent lignin, TDN: total digestible nutrients, NEm: net energy for maintenance, NEg: net energy for growth, NFC: non-fiber carbohydrate, CA: carbohydrate A fraction; ethanol soluble carbohydrates, CB1: carbohydrate B1 fraction; starch, CB2: carbohydrate B2 fraction; soluble fiber, CB3: carbohydrate B3 fraction; available insoluble fiber, CC: carbohydrate C fraction; unavailable carbohydrate, PA+B1: protein A and B1 fractions; soluble CP, PB2: protein B2 fraction; intermediate degradable CP, PB3: protein B3 fraction; slowly degradable fiber-bound CP, PC: protein C fraction; unavailable CP.
