# Supplementary material for: Repeatability of feed efficiency and its relationship with carcass traits in Hanwoo steers during their entire growing and fattening period
Source: Anim Biosci. 2024 Apr 25;37(9):1568–80. doi: 10.5713/ab.24.0074 (PMC11366531; doi:10.5713/ab.24.0074)
Supplement: Supplementary file 7 [file ab-24-0074-Supplementary-Table-7.pdf]

Supplementary Table 7. Diet composition (g/kg DM or as stated) of the concentrate mixes in fattening period 2

Items[2]	Treatment[1]	
	Commercial	CLS
Corn, flaked	240	250
Wheat, ground	87	74
Corn, ground	0	25
Lupin, flaked	40	40
Coconut oil meal	70	0
DDGS	54	102
Soybean meal	20	0
Rapeseed meal	30	17
Palm kernel meal	80	100
Wheat flour	70	82
Corn gluten feed	159	200
Wheat bran	52	0
Beet pulp pellet	20	20
Limestone	25	27
Molasses	25	20
CMS	15	15
CSL	0	15
Salt	7	5
Sodium bicarbonate	3	3
Vitamin and mineral mix[3]	3	6

[1]CSL, Corn steep liquor

[2]DDGS, Distillers dried grains; CMS, Condensed molasses solubles; CSL, Corn steep liquor.

[3]33,330,000 IU/kg vitamin A, 40,000,000 IU/kg vitamin D, 20.86 IU/kg vitamin E, 20 mg/kg Cu, 90 mg/kg Mn, 100 mg/kg Zn, 250 mg/kg Fe, 0.4 mg/kg I, and 0.4 mg/kg Se.
