# Supplementary material for: Repeatability of feed efficiency and its relationship with carcass traits in Hanwoo steers during their entire growing and fattening period
Source: Anim Biosci. 2024 Apr 25;37(9):1568–80. doi: 10.5713/ab.24.0074 (PMC11366531; doi:10.5713/ab.24.0074)
Supplement: Supplementary file 8 [file ab-24-0074-Supplementary-Table-8.pdf]

**Supplementary Table 8.** Analyzed chemical composition (g/kg DM or as stated) of the feeds in fattening period 2

| Items[1] | Treatment[1] Commercial | CSL | Annual ryegrass |
|---|---|---|---|
| DM, g/kg as fed | 878 | 865 | 900 |
| OM | 912 | 922 | 936 |
| CP | 175 | 179 | 56 |
| SOLP | 58 | 61 | 20 |
| NDICP | 25 | 25 | 15 |
| ADICP | 13 | 14 | 12 |
| aNDF | 301 | 311 | 739 |
| ADF | 149 | 146 | 517 |
| ADL | 35 | 32 | 82 |
| Ether extract | 44 | 44 | 8 |
| Ash | 88 | 79 | 64 |
| Ca | 14 | 13 | 5 |
| P | 6 | 6 | 1 |
| K | 10 | 10 | 8 |
| Na | 4 | 4 | 4 |
| Cl | 8 | 7 | 3 |
| S | 3 | 3 | 2 |
| Mg | 4 | 3 | 1 |
| TDN | 727 | 738 | 489 |
| NEm, MJ/kg DM | 7.1 | 7.3 | 3.9 |
| NEg, MJ/kg DM | 4.5 | 4.6 | 1.6 |
| Total carbohydrates | 692 | 699 | 872 |
| NFC | 416 | 413 | 147 |
| Carbohydrate fraction, g/kg carbohydrate | | | |
| CA | 72 | 50 | 42 |
| CB1 | 449 | 492 | 11 |
| CB2 | 80 | 49 | 116 |
| CB3 | 279 | 301 | 604 |
| CC | 121 | 109 | 226 |
| Protein fraction, g/kg CP | | | |
| PA+B1 | 328 | 341 | 359 |
| PB2 | 529 | 520 | 369 |
| PB3 | 66 | 64 | 52 |
| PC | 76 | 75 | 221 |

[1]CSL, Corn steep liquor

[2]DM: dry matter, OM: organic matter, CP: crude protein, SOLP: soluble CP, NDICP: neutral detergent insoluble CP, ADICP: acid detergent insoluble CP, aNDF: neutral detergent fiber analyzed using a heat stable amylase and expressed inclusive of residual ash, ADF: acid detergent fiber, ADL: acid detergent lignin, TDN: total digestible nutrients, NEm: net energy for maintenance, NEg: net energy for growth, NFC: non-fiber carbohydrate, CA: carbohydrate A fraction; ethanol soluble carbohydrates, CB1: carbohydrate B1 fraction; starch, CB2: carbohydrate B2 fraction; soluble fiber, CB3: carbohydrate B3 fraction; available insoluble fiber, CC: carbohydrate C fraction; unavailable carbohydrate, PA+B1: protein A and B1 fractions; soluble CP, PB2: protein B2 fraction; intermediate degradable CP, PB3: protein B3 fraction; slowly degradable fiber-bound CP, PC: protein C fraction; unavailable CP.
