# Supplementary material for: Repeatability of feed efficiency and its relationship with carcass traits in Hanwoo steers during their entire growing and fattening period
Source: Anim Biosci. 2024 Apr 25;37(9):1568–80. doi: 10.5713/ab.24.0074 (PMC11366531; doi:10.5713/ab.24.0074)
Supplement: Supplementary file 9 [file ab-24-0074-Supplementary-Table-9.pdf]

Supplementary Table 9. Diet composition (g/kg DM or as stated) of the concentrate mixes in fattening period 3

	Treatment	
Items[1]	Commercial	High strach
Corn, flaked	290	292
Corn, ground	30	114
Wheat, ground	60	69
Barley, flaked	41	41
Lupin, flaked	21	21
Wheat flour	41	13
Corn gluten feed	207	147
Palm kernel meal	103	93
Rapeseed meal	11	41
Corn germ meal	30	0
DDGS	0	20
Cottonseed, whole	30	30
Rice bran	30	30
Mixed hull	32	14
Limestone	30	31
Molasses	18	18
CMS	11	11
CSL	3	3
Salt	7	8
Sodium bicarbonate	3	3
Vitamin and mineral mix[2]	3	3

[1]DDGS, Distillers dried grains; CMS, Condensed molasses solubles; CSL, Corn steep liquor.
[2]33,330,000 IU/kg vitamin A, 40,000,000 IU/kg vitamin D, 20.86 IU/kg vitamin E, 20 mg/kg Cu, 90 mg/kg Mn, 100 mg/kg Zn, 250 mg/kg Fe, 0.4 mg/kg I, and 0.4 mg/kg Se.
