# Supplementary material for: Repeatability of feed efficiency and its relationship with carcass traits in Hanwoo steers during their entire growing and fattening period
Source: Anim Biosci. 2024 Apr 25;37(9):1568–80. doi: 10.5713/ab.24.0074 (PMC11366531; doi:10.5713/ab.24.0074)
Supplement: Supplementary file 10 [file ab-24-0074-Supplementary-Table-10.pdf]

Supplementary Table 10. Analyzed chemical composition (g/kg DM or as stated) of the feeds in fattening period 3

Items[1]	Treatment		Annual ryegrass
	Commercial	High starch	
DM, g/kg as fed	888	883	900
OM	919	918	936
CP	150	154	56
SOLP	53	53	20
NDICP	22	20	15
ADICP	12	12	12
aNDF	283	273	739
ADF	155	138	517
ADL	45	40	82
Ether extract	44	47	8
Ash	81	82	64
Ca	13	14	5
P	6	6	1
K	10	9	8
Na	4	5	4
Cl	7	8	3
S	3	3	2
Mg	3	3	1
TDN	715	728	489
NEm, MJ/kg DM	7.3	7.5	3.9
NEg, MJ/kg DM	4.7	4.9	1.6
Total carbohydrates	725	717	872
NFC	464	464	147
Carbohydrate fraction, g/kg carbohydrate			
CA	64	62	42
CB1	509	540	11
CB2	67	45	116
CB3	212	220	604
CC	148	133	226
Protein fraction, g/kg CP			
PA+B1	352	348	359
PB2	503	522	369
PB3	61	51	52
PC	83	79	221

[1]DM: dry matter, OM: organic matter, CP: crude protein, SOLP: soluble CP, NDICP: neutral detergent insoluble CP, ADICP: acid detergent insoluble CP, aNDF: neutral detergent fiber analyzed using a heat stable amylase and expressed inclusive of residual ash, ADF: acid detergent fiber, ADL: acid detergent lignin, TDN: total digestible nutrients, NEm: net energy for maintenance, NEg: net energy for growth, NFC: non-fiber carbohydrate, CA: carbohydrate A fraction; ethanol soluble carbohydrates, CB1: carbohydrate B1 fraction; starch, CB2: carbohydrate B2 fraction; soluble fiber, CB3: carbohydrate B3 fraction; available insoluble fiber, CC: carbohydrate C fraction; unavailable carbohydrate, PA+B1: protein A and B1 fractions; soluble CP, PB2: protein B2 fraction; intermediate degradable CP, PB3: protein B3 fraction; slowly degradable fiber-bound CP, PC: protein C fraction; unavailable CP.
